# Initial characterization of ruminal T lymphocytes in lactating dairy cows

**DOI:** 10.3168/jdsc.2025-0838

**Published:** 2025-10-10

**Authors:** Lolita Vandevoorde, Kirby Krogstad

**Affiliations:** Department of Animal Sciences, The Ohio State University, Wooster, OH 44961

## Abstract

γδ T cells may regulate immune responses through interleukin-10 secretion and reduce cytotoxic and helper T cell proliferation, in vitro. γδ T cells are prominent in ruminant peripheral blood, but their presence in a recently defined ruminal immune cell population remains unclear. Our study aimed to determine the relative proportions of T cell types within rumen tissue (heterogeneous tissue consisting of lamina propria, stratified epithelium, and vasculature) of healthy dairy cows through spectral flow cytometry, and we hypothesized that the largest proportion of ruminal T cells would be γδ T cells. We used spectral flow cytometry to assess ruminal and peripheral blood T cell proportions. The panel included CD45 (leukocytes), CD3 (T lymphocytes), CD4 (helper T cells), and TCRN-24 (γδ T cells). Results were analyzed with a mixed linear model that included the effect of blood versus rumen tissue and the random effect of cow. Cows were clinically healthy throughout the experiment, as indicated by their DMI (26.0 ± 3.44 kg/d), milk yield (50.6 ± 12.99 kg/d; mean ± SD), and ruminal pH (6.06 ± 0.55). CD3+ cells constituted 83.9% ± 2.85% (mean ± SEM) of ruminal CD45+ cells compared with 3.3% ± 2.85% of CD45+ cells in blood. On a CD3+ basis, TCRN24+ were the most prominent subset of T lymphocytes in rumen tissue samples and were enriched compared with blood (78.7% ± 6.92% of CD3+ vs. 38.1 ± 6.92% of CD3+). The CD4+ were reduced as a proportion of T cells in the rumen compared with blood (1.4% ± 1.95% of CD3+ vs. 21.4 ± 1.95% of CD3+). We observed a TCRN24+CD4+ population isolated from the rumen which was lesser in blood (9.0% ± 2.72% of CD3+ vs. 1.5 ± 2.72% of CD3+). We confirmed our hypothesis that γδ T cells are the most prominent proportion of T cells within the rumen tissue in healthy lactating dairy cows. Furthermore, we observed a unique population that was both TCRN24+ and CD4+; investigations of this potential T cell subtype are needed to confirm their presence and determine their function. Understanding the role of T lymphocytes in rumen immunity may guide strategies that strengthen rumen health and digestion while reducing gut-derived inflammation.

## Highlights

• T lymphocytes are the most prominent leukocytes in the rumen tissue.
• γδ T cells are the most prominent T-cell subset in the rumen tissue.
• A novel cell type (CD4+TCRN24+) may exist within the rumen.

## Graphical Abstract

**Figure:**
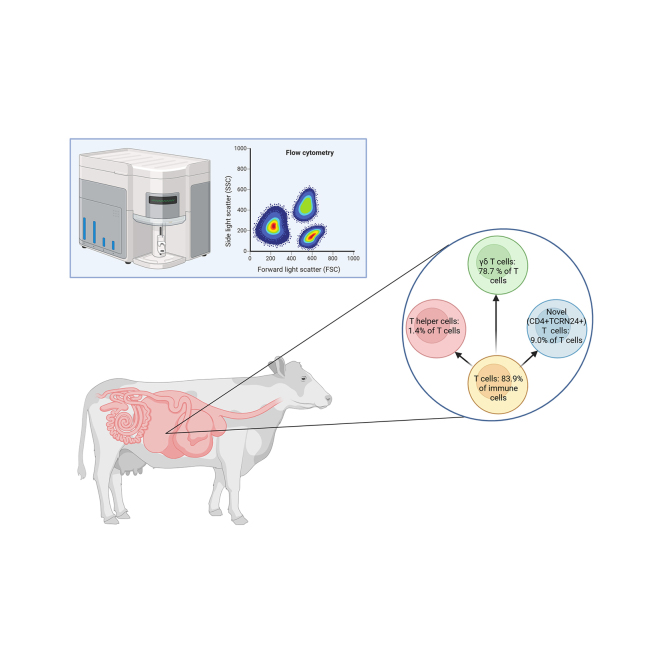
**Summary:** Our study aimed to determine the relative proportions of T cell types in the rumen tissue of healthy dairy cows through spectral flow cytometry, hypothesizing that the largest proportion would be γδ T cells. We used spectral flow cytometry to assess ruminal T cell proportions. The panel included CD45+ (leukocytes), CD3+ (T lymphocytes), CD4+ (helper T cells), and TCRN-24+ (γδ T cells). CD3+ cells constituted 83.9% ± 2.85% (mean ± standard error of the mean) of ruminal CD45+ cells. On a CD3+ basis, TCRN24+ cells were the most prominent subset of T lymphocytes in rumen tissue (78.7% ± 6.92% of CD3+). CD4+ were reduced as a proportion of T cells in the rumen (1.4% ± 1.95% of CD3+). We observed a TCRN24+CD4+ population in the rumen (9.0% ± 2.72% of CD3+). Understanding the role of T lymphocytes in rumen immunity may guide strategies that strengthen rumen health and digestion while reducing gut-derived inflammation. Created in BioRender. Krogstad, K. (2025) https://BioRender.com/amyibh6.

Rumen and gastrointestinal health are increasingly discussed but lack clear definition. Rumen health in cattle is frequently identified through the occurrence of SARA. Thus, evaluating reticulorumen pH is common in research and used to discuss and measure “rumen health” (Humer et al., 2018). Reticulorumen pH is also being measured more on commercial dairy farms with the introduction of pH boluses (Mazon et al., 2020) that allow for instantaneous measurement of reticulorumen pH in large numbers of cows. However, rumen and gastrointestinal pH alone are not indicators of rumen health; reduced rumen or gastrointestinal pH are not always accompanied by reduced milk production or poorer health status (Abeyta et al., 2023; Krogstad et al., 2025). Perhaps the immune cells of the rumen offer a better view of rumen and gut health in cattle and thus require deeper understanding. By gaining knowledge of ruminal immune cells, we may be able to create feeding or management programs that improve animal health, production, and efficiency.

A recent study characterized ruminal immune cells in healthy lactating dairy cattle (Krogstad et al., 2025). Researchers observed that the main immune cells present within rumen tissue were T cells; T cells composed 85% of viable immune cells observed in the rumen tissue, with the proportion of cells unaffected by a mild SARA-induction diet. The majority of these cells were present within the basal layers of the rumen epithelium or within the lamina propria. The location and resident or transient nature of these ruminal immune cells is a gap in our knowledge. That study also observed that 7% of cells were of myeloid origin, with the remaining undefined (Krogstad et al., 2025). Their data align with previous evidence suggesting that ruminal immune cells exist, with T cells or T cell markers being observed through single-cell RNA sequencing or immunohistochemistry approaches (Jonsson et al., 2020; Wu et al., 2022). There are currently no investigations of specific T cell phenotypes within the rumen of lactating dairy cattle. Understanding what types of T cells exist in a healthy rumen is vital, as ruminal T cells likely complete important tasks that help maintain rumen tissue barrier health and function.

The two T cell subsets largely discussed within literature are cytotoxic T cells and helper T cells (Andersen et al., 2006); cytotoxic T cells (CD8+) kill infected cells (Andersen et al., 2006) through perforin and granzyme secretion (Hay and Slansky, 2022). Helper T cells (CD4+) recognize antigens and secrete cytokines targeting the activation of other immune cells (Kandel et al., 2021). Another T cell subset is γδ T cells (TCR-N24+), which are prominent in ruminants (Hope, 2016). One study demonstrated that γδ T cells engage in immune regulation in ruminant peripheral blood by reducing proliferation of CD4+ and CD8+ T cells in vitro, along with secreted IL-10 (Guzman et al., 2014). Additionally, γδ T cells proliferate in response to IL-10 and TGF-β (Hope, 2016). IL-10 has strong anti-inflammatory properties and helps limit excessive pathological immune responses, preventing damage to the host (Iyer and Cheng, 2012). These functions may not hold for all γδ T cells, though, as skin-draining γδ T cells could not be induced to produce IL-10 (Van Rhijn et al., 2007). Therefore, our current understanding is that some γδ T cells have regulatory functions in cattle through IL-10 secretion and reducing proliferation of T-helper and cytotoxic T cells. We postulate that γδ T cells are prominent in rumen tissue and may have a similar regulatory function within the rumen tissue of dairy cattle.

The objective of our study was to characterize subsets of T lymphocytes from rumen tissue samples of healthy lactating dairy cows. We hypothesized that the most prominent proportion of T cells would be γδ T cells.

We conducted an observational study using 4 ruminally cannulated lactating Holstein cows housed at the Krauss Dairy Facility in Wooster, Ohio. The cows were enrolled in 2 cohorts; the first had 3 cannulated cows, and the second had 1 cow. Selection for this study was based solely on availability of lactating cannulated cows at that time. The first cohort of cows (n = 3) were housed in individual tiestalls with ad libitum access to feed and water for 14 d. The final cow was housed in a tiestall for 1 d to obtain an additional rumen and blood sample for immune cell analysis. The cows were fed once per day at 0800 h and milked twice daily at 0400 and 1600 h. The TMR fed to the cows was balanced to meet nutrient requirements of lactating cows producing 40 kg/d of milk (NASEM, 2021).

The TMR samples were collected once per week throughout the experiment and composited before nutrient analysis. The TMR samples were analyzed for DM (AOAC International, 2000), CP (N × 6.25; Flash 2000, Thermo Fisher Scientific), NDF (Van Soest et al., 1991), ash (AOAC International, 2000), and particle size using a Penn State Particle Separator (Kononoff et al., 2003). Milk samples were collected at both milkings on d 2 and 9 of the experiment for analysis of milk composition, MUN, and SCC by Fourier-transform infrared spectroscopy and flow cytometry, respectively (DHI Cooperative, Columbus, OH). Body weight and BCS were measured after feeding on d 2 and 9. Rumen pH was measured with a handheld pH probe on d 2 and 11 every 4 h for 24 h beginning at feeding (Krogstad et al., 2024).

Partial rumen evacuations were conducted on d 14 before feeding to facilitate rumen tissue harvest as described previously (Krogstad et al., 2025). Approximately 1.5 g (wet basis) of rumen tissue was snipped from the rumen using surgical scissors and rinsed 3 times in Hanks' balanced salt solution (**HBSS**) with 1% penicillin and streptomycin. The samples were then transported on ice to the laboratory for further processing. The ruminal contents were placed back into the rumen upon completion of rumen tissue harvest. Blood samples for flow cytometric analysis were taken via coccygeal venipuncture immediately after the rumen evacuations into tubes containing K_2_EDTA (Krogstad et al., 2025).

Peripheral blood immune cells were isolated as described previously (Krogstad et al., 2024). In short, blood samples were centrifuged for 15 min at 1,500 × *g* at 4°C to separate and remove the plasma layer. Then, 5 mL of cold Ca++Mg++-free Dulbecco's PBS (**DPBS**) was added, and the sample was centrifuged again at 1,500 × *g* at 4°C for 10 min. The supernatant was pipetted off, and we then used ammonium-chloride-potassium lysis buffer and incubated the cells at 4°C in the dark for 8 min to lyse red blood cells. The sample was centrifuged for 5 min at 400 × *g* at 4°C, and the supernatant was removed. The cells were then suspended in DPBS and centrifuged. This step was repeated once more (Krogstad et al., 2025). After the second washing step, cells were suspended in DPBS before staining.

Ruminal immune cells were isolated according to the procedure previously described (Krogstad et al., 2025). Briefly, rumen tissue samples were minced with a scalpel and digested for 1 h at 39°C in a digestion solution containing prewarmed 1640 RPMI supplemented with 2% fetal bovine serum (**FBS**), collagenase type VIII, DNase I, liberase, and HEPES. The samples were filtered twice through 100-µm and 70-µm filters, respectively. The cells were then centrifuged at 400 × *g* for 5 min at 4°C and cleaned with HBSS twice before cell staining.

The rumen and blood immune cells were stained according to the previously described procedure (Krogstad et al., 2024). Briefly, the cells were stained with Zombie Yellow (Biolegend; cat. no. 423104) and incubated in the dark on ice for 20 min. Following incubation, DPBS with 1% FBS (**FACS**) was added, cells were centrifuged for 5 min at 400 × *g* at 4°C, and the supernatant was removed. Additional FACS buffer was added to each sample, and the cells were incubated in the dark on ice for 10 min to facilitate Fc blocking. An antibody cocktail was added to each sample and incubated in the dark for 30 min. The antibody cocktail included CD45, a marker for all leukocytes, conjugated to fluorescein isothiocyanate (Bio-Rad; cat no. MCA832F). The CD45 antibody was incubated with the cells at an estimated concentration of 5.0 µg/mL. The CD3 antibody, a marker for all T lymphocytes (MM1A; Washington State Monoclonal Antibody Center), was conjugated to PE-Cy5 (Abcam; cat no. ab102893), with incubation concentration of 20.8 µg/mL. CD4, conjugated to Pacific Blue (Bio-Rad; cat no. MCA1653PB), was used to identify helper T cells. It was included at 5.0 µg/mL in the final incubation. Finally, the TCRN24 antibody (GB21A; Washington State Monoclonal Antibody Center) was conjugated to AlexaFluor-647 (Abcam; cat no. ab269823) and used to detect γδ T cells. This antibody was included at 20.8 µg/mL for incubation. After staining, cells were washed once more using FACS buffer and resuspended in fixation buffer (DPBS with 1% paraformaldehyde) for 15 min in the dark. The sample was then centrifuged at 5 min at 400 × *g* at 4°C, the supernatant was removed, and the cells were resuspended in FACS and analyzed on a Northern Lights Flow Cytometer (Cytek). The gating strategy is displayed in Figure 1. All gates were set using fluorescence minus one controls.

**Figure 1:**
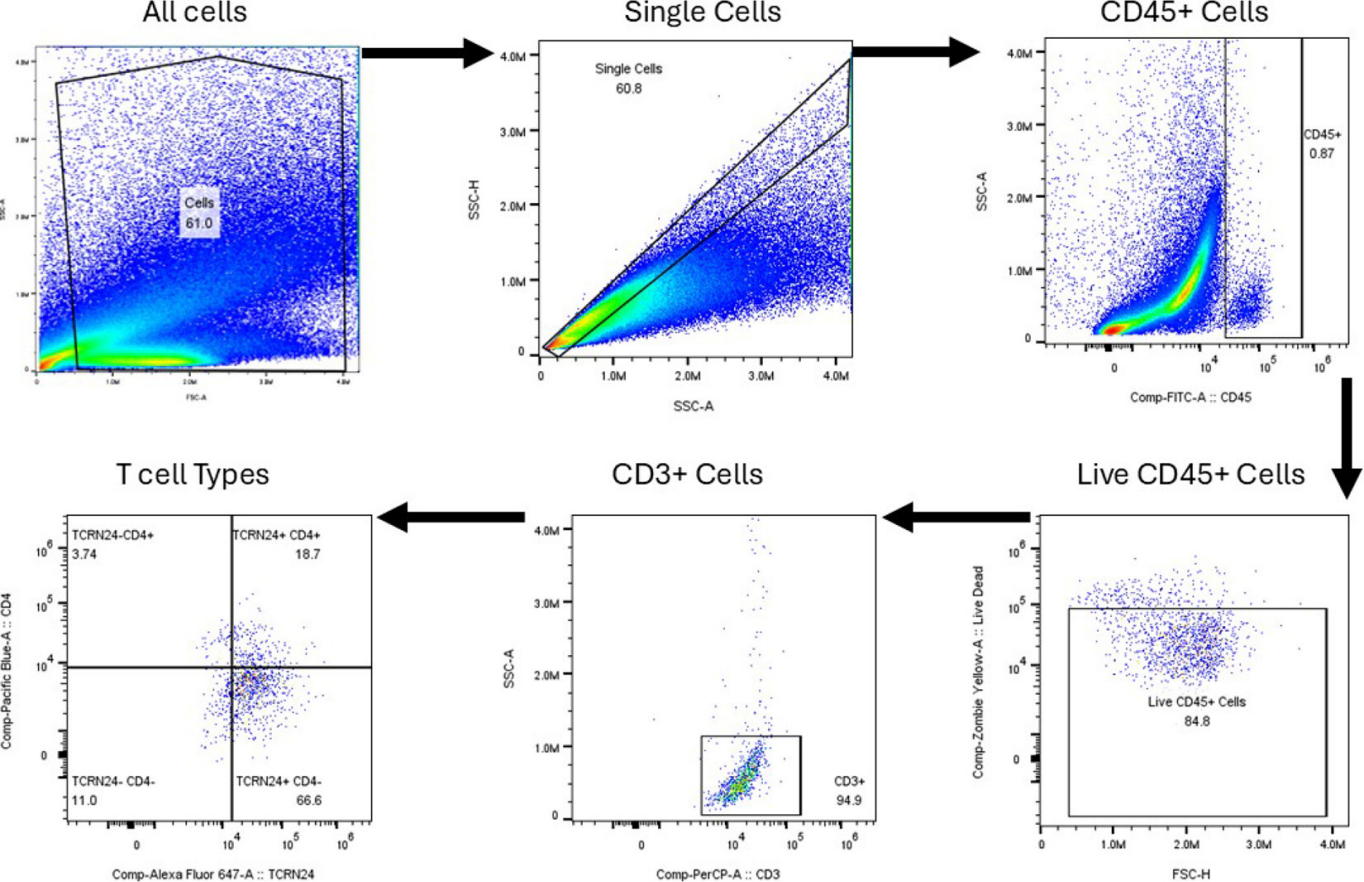
Gating strategy used for spectral flow cytometry for evaluating peripheral blood and ruminal immune cells in lactating dairy cattle. First, debris was removed; then we gated for singlet cells. We then gated for CD45+ cells, followed by gating for viable cells using a fixable live-dead stain. Then we gated for T cells and T cell subtypes.

Feed intake, milk yield, milk components, and feed analysis were summarized in Microsoft Excel (version 16.7). Flow cytometry data were analyzed using a linear mixed model in SAS (v. 9.4; SAS Institute Inc.). The fixed effect was source (rumen vs. blood), and the random effect was cow. Statistical significance was declared when *P* ≤ 0.05, and tendencies are discussed at *P* ≤ 0.10.

The lactating dairy cows were clinically healthy throughout the experimental period. Body weight was 751 ± 91.5 kg, and BCS was 2.81 ± 0.41 (mean ± SD). Dry matter intake averaged 26.0 ± 3.44 kg/d, and milk yield was 50.6 ± 12.99 kg/d.

Rumen pH was within normal ranges and followed a normal diurnal pattern for dairy cattle fed once daily in a tiestall setting (Krogstad et al., 2025). Rumen pH averaged 6.1 ± 0.55, with minimum and maximum rumen pH of 5.26 ± 0.142 and 6.95 ± 0.128, respectively (Figure 2). The TMR was 51.1% ± 3.44% DM, 7.6% ± 0.47% ash, 34.4% ± 2.98% NDF, and 15.4% ± 0.36% CP (mean ± SD). As-fed particle size distribution for the TMR was 10.3% ± 2.02% retained on the 19-mm sieve, 38.5% ± 0.79% retained on the 8-mm sieve, 14.0% ± 2.10% retained on the 4-mm sieve, and 37.3% ± 0.71% retained in the pan.

**Figure 2:**
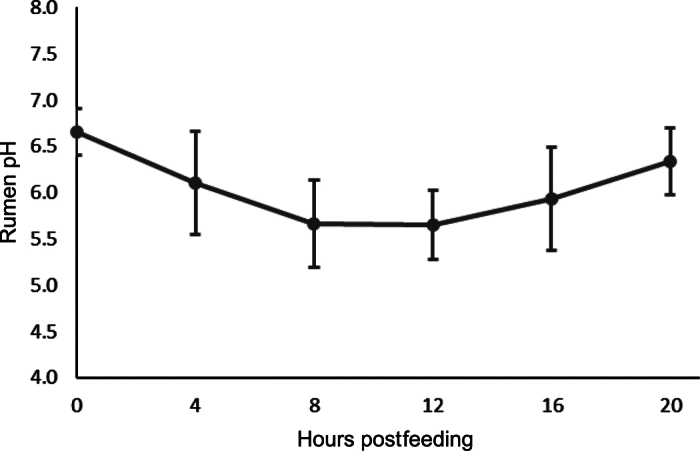
Mean rumen pH (±SD) for lactating dairy cows evaluated for ruminal immune cell populations.

The CD3+ T cells were 83.9% ± 2.85% (mean ± SEM) of viable immune cells from the rumen tissue samples (% CD45+), significantly higher than their proportion in peripheral blood (3.3% ± 2.85% CD45+; *P* < 0.01; Figure 3). The proportion of helper T cells (CD3+CD4+) was not enriched in rumen tissue samples compared with peripheral blood (0.7% ± 0.60% vs. 1.3% ± 0.60% CD45+; *P* = 0.55). The γδ T cells were enriched in the rumen (65.5% ± 0.78% of CD45+) compared with peripheral blood (1.2% ± 0.78% of CD45+; *P* < 0.01). A unique TCRN24+CD4+ subset was identified in rumen tissue samples (7.8% ± 2.59% of CD45+) and tended to be enriched compared with peripheral blood (0.0% ± 2.59% of CD45+; *P* = 0.08).

**Figure 3:**
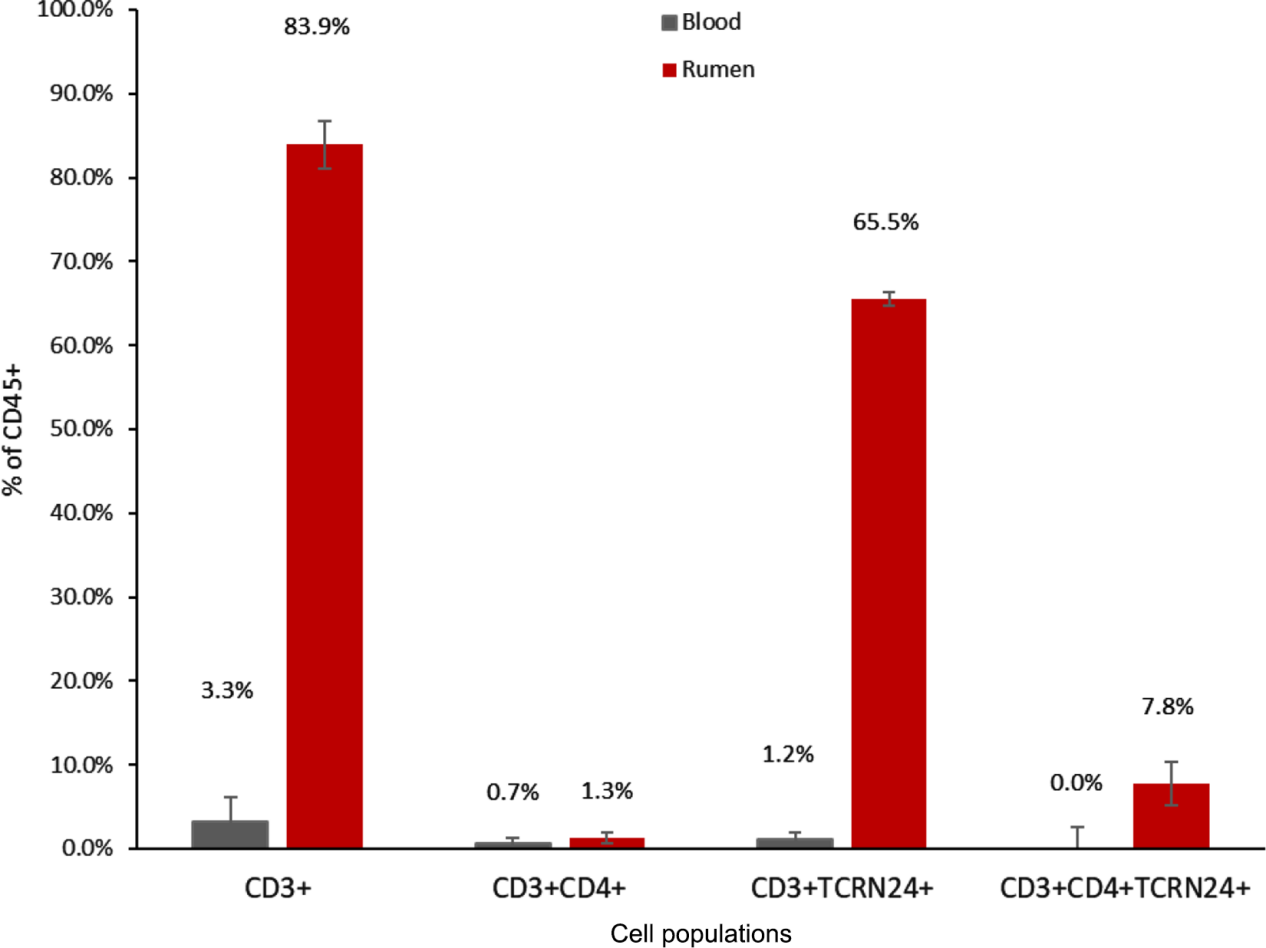
Flow cytometry results for lactating dairy cows evaluated for ruminal immune cell populations. Error bars reflect SEM. The rumen had greater proportions of CD45+ versus CD3+ cells than blood (*P* < 0.01), greater proportions of CD3+TCRN24+ (*P* < 0.01), and tended to have greater CD3+TCRN24+CD4+ (*P* = 0.08). The proportion of CD3+CD4+ was not different for blood versus rumen (*P* = 0.55).

Relative to CD3+ cells, we observed that CD4+ T cells were reduced in rumen tissue (1.4% ± 1.95% of CD3+) compared with peripheral blood (21.4% ± 1.95% of CD3+; *P* < 0.01). The TCRN24+ cells constituted 78.7% ± 6.92% of ruminal CD3+ cells, significantly greater than their proportion in peripheral blood (38.1% ± 6.92% of CD3+; *P* < 0.01). The unique TCRN24+CD4+ subset tended to be greater in rumen tissue (9.0% ± 2.72% of CD3+) than peripheral blood (1.5% ± 2.72% of CD3+, *P* = 0.10).

The DMI, milk yield, rumen pH, and the cow's general appearance were normal, indicating that cows were clinically healthy throughout the observation period. Thus, our experiment confirms that leukocytes inhabit rumen tissue samples from healthy lactating dairy cows. Also, we confirmed that ruminal T cells are the most abundant immune cells in rumen tissue samples of healthy lactating dairy cows, which is in line with Krogstad et al. (2025) who demonstrated that T cells were ∼85% of ruminal leukocytes. We still are limited in our understanding of whether these cells reside within the rumen epithelium or are transient. Future investigations should work to confirm the localization of these cells within the complex ruminal architecture. Our results also align with previous investigations using single-cell RNaseq and immunohistochemistry that suggested T cells were prominent in rumen tissue of cattle and sheep (Jonsson et al., 2020; Huang et al., 2023); although neither definitively proves localization of these cells within the rumen epithelium, they do support the hypothesis that T cells are important in the context of the rumen, whether by resident cells or by increased trafficking to the ruminal site.

Our experiment establishes baseline insights into specific T cell populations that reside or transiently exist within the rumen tissue of healthy lactating dairy cows. T cells have also been observed in adipose tissue (Contreras et al., 2016), demonstrating that T cells are prominent in other tissues in addition to the rumen. Our results agree with the hypothesis that γδ T cells are the predominant T cell subset in rumen tissue and align with the broader literature demonstrating that γδ T cells are a substantial portion of T lymphocytes in ruminants (Hope, 2016). Furthermore, it adds to the locations of γδ T cells in cattle; previous investigations have identified their presence in spleen, adipose, and intestinal tissues of bovines (MacHugh et al., 1997; Oliveira et al., 2019; Cangiano et al., 2024). The γδ T cells also play a major role in skin of bovines, but these skin-draining γδ T cells do not produce IL-10, IFN-γ, or TNF-α, which demonstrates that site-specific functions may exist (Van Rhijn et al., 2007). Previous single-cell RNaseq analysis suggested that T-helper, especially T-helper 17 cells, were prominent within rumen tissue of male lambs within the first 25 d of life (Huang et al., 2023). Our results suggest T-helper cells (CD4+) may be less prominent in mature ruminants based on cell proportions. Follow-up investigations should identify whether the CD4+ cells we observed were T-helper 17 or some other T-helper cell lineage. Perhaps species-specific differences may exist between ruminants, or perhaps the immune cell populations present in rumen tissue change as ruminants mature. Additional investigations across species and ages may yield insights into rumen development across species.

The significantly higher proportion of γδ T cells in the rumen of lactating dairy cattle compared with blood suggests a unique immunophenotype within the rumen, where γδ T cells may play a substantial role in maintaining tissue immunity and integrity. Perhaps γδ T cells regulate gastrointestinal inflammation through IL-10 secretion, an important function of γδ T cells (Iyer and Cheng, 2012; Guzman et al., 2014). Previous research suggests that γδ T cell functions likely differ across tissues, which increases the need for context-dependent investigations of immune cell function (Van Rhijn et al., 2007). The lesser proportion of CD4+ cells in ruminal tissue may be due to γδ T cells' ability to reduce CD4+ T cell proliferation (Guzman et al., 2014; Hope, 2016), but that is unlikely because CD4+ and γδ T cells are both present in adipose tissue (Oliveira et al., 2019). Perhaps CD4+ simply traffic less to the rumen tissue than other locations.

One limitation of our analysis is that we did not investigate an additional T cell subset, CD8+ T cells. Future research should investigate the proportion of CD8+ T cells within the rumen. CD8+ T cells have been observed in adipose tissue and corpus luteum of dairy cattle, which suggests they are able to traffic to or reside in other tissues (Poole and Pate, 2012; Oliveira et al., 2019). Nonimmune cells should also be investigated as they play a role in immune function and activation (Huang et al., 2023). Investigating both immune and nonimmune cells will provide a more complete picture of rumen tissue inflammation and immune function in dairy cattle.

The observed presence of the unique TCRN24+CD4+ T cell subset in rumen tissue is intriguing, as it suggests that an uncharacterized population of helper-like γδ T cells in dairy cows may exist. We are not aware of this specific cell type having been demonstrated previously. These cells may contribute to immunoregulatory functions by secreting a wide variety of cytokines, which may be highly context-dependent. As previously mentioned, CD4+ cells transmit signals via cytokine release that enhance function of other immune cells (Kandel et al., 2021), and TCRN24+ cells have a variety of functions including the secretion of cytokines (Hope, 2016). We speculate that this novel cell type may have the ability to release cytokines produced by both T cell subtypes and thus have a variety of effects within rumen tissue. Unique variants of γδ T cells have been previously discussed. For example, a subpopulation of γδ T cells in humans has been observed to express CD8 (Davis et al., 1996). Thus, different variants of γδ T cells likely exist in bovines and may provide a wide array of functions to help maintain the ruminal barrier (Andersen et al., 2006).

Our results demonstrate that γδ T cells are the predominant subset of T lymphocytes in rumen tissue samples, which supports our hypothesis. We cannot unequivocally say whether these cells reside in the rumen epithelium or lamina propria or whether they are resident or transient. The addition of a TCRN24+CD4+ populations suggests that a unique immune cell profile may exist in the rumen and that further investigation is needed to determine their function. Also, the addition of other T cell types, such as CD8+ cells, may provide a more complete picture of the ruminal immune cell dynamics. Nonetheless, our initial findings provide a baseline for understanding immune cell dynamics in the rumen and can provide foundational knowledge for future research, which should include investigating immune cell functions such as cytokine secretion, and exploring immune dynamics in diseased states, where immune dysregulation and altered immune cell proportions may contribute to the systemic inflammation observed during gastrointestinal stress. Also, investigations across species or ages may yield insights into rumen development, which may aid in optimizing management strategies for livestock.
